# RETRACTED: Qadir et al. Synthesis and Characterization of Metal Particles Using Malic Acid-Derived Polyamides, Polyhydrazides, and Hydrazides. *Molecules* 2025, *30*, 134

**DOI:** 10.3390/molecules31101763

**Published:** 2026-05-21

**Authors:** Muhammad Farhan Qadir, Somavia Ameen, Rida Fatima, Nadim Ullah, Gamal A. Shazly, Abu Summama Sadavi Bilal, Mehwish Nazar, Anoosha Sajjad, Tawaf Ali Shah, Yukun Yang

**Affiliations:** 1 School of Life Science, Shanxi University, Taiyuan 030006, China; g207@email.sxu.edu.cn; 2 Department of Chemistry, School of Science, Tianjin Key Laboratory of Molecular Optoelectronic Science, Tianjin University, Tianjin 300072, China; 3 Institute of Chemical Sciences, Bahauddin Zakariya University, Multan 60000, Pakistan; 4 Institute of Chemical Sciences, University of Peshawar, Peshawar 25120, Pakistan; 5 Department of Pharmaceutics, College of Pharmacy, King Saud University, Riyadh 11451, Saudi Arabia; 6 Department of Mechanical Engineering, University of Engineering and Technology, Taxila 47080, Pakistan; 7 Department of Chemistry, School of Science, University of Management and Technology, Lahore 42000, Pakistan; 8 Chemistry Department, Islamia College University, Peshawar 25000, Pakistan; 9 College of Agriculture Engineering and Food Sciences, Shandong University of Technology, Zibo 255000, China

The journal retracts the article titled “Synthesis and Characterization of Metal Particles Using Malic Acid-Derived Polyamides, Polyhydrazides, and Hydrazides” [1], cited above.

Following publication, concerns regarding the authorship and image irregularities in this published article [1] were brought to the attention of the Editorial Office.

In accordance with standard journal procedures, the Editorial Office and Editorial Board conducted an investigation that confirmed indications of inappropriate image editing in Figures 1b, 2e and 3b,c. Although the authors cooperated with the investigation, they could not provide supporting materials for the Editorial Board to evaluate and validate the study’s origin. As a result, the Editorial Board has lost confidence in the reliability of the findings and results and has decided to retract this paper as per MDPI’s retraction policy.

This retraction was approved by the Editor-in-Chief of the journal *Molecules*.

The corresponding author requested the retraction of the article and agrees with the decision of the Editorial Board. He would like to express his apologies to the editors and readers. The author states that the hard drive storing the study’s original experimental data suffered an unrecoverable hardware failure. The remaining authors did not provide any comment.
